# Monkeypox Presenting as Periorbital Cellulitis: A Case Report

**DOI:** 10.7759/cureus.65517

**Published:** 2024-07-27

**Authors:** Unaiza Batool, Fahad S Siddiqui, Jessika Sanz, Shoaib Siddiqui

**Affiliations:** 1 Infectious Disease, Alabama College of Osteopathic Medicine, Dothan, USA; 2 Dermatology, Kansas City University-Graduate Medical Education Consortium/Advanced Dermatology and Cosmetic Surgery, Maitland, USA; 3 Internal Medicine, Hospital Corporation of America (HCA) Florida Osceola Hospital, Kissimmee, USA; 4 Infectious Disease, Hospital Corporation of America (HCA) Florida Osceola Hospital, Kissimmee, USA

**Keywords:** dermatology, infectious disease, case report, periorbital cellulitis, monkeypox

## Abstract

Monkeypox, a viral disease caused by the monkeypox virus is well known for its characteristic rash with macular, papular, and vesicular stages. Although the skin is one of the most affected organs by monkeypox, the virus can also impact the respiratory, ophthalmologic, genitourinary, and gastrointestinal systems, among others. It is extremely common for the disease to begin with flu-like symptoms in the prodromal phase before cutaneous manifestations emerge. Here, we describe a unique case of monkeypox infection in which a patient first presented with periorbital cellulitis before any characteristic skin findings appeared. The source of the infection was unknown and the patient recovered without any complications to date. This odd presentation of monkeypox reiterates the need for a detailed evaluation of patients with a similar presentation.

## Introduction

Monkeypox, a viral zoonotic disease affecting humans that was previously considered endemic only to sub-Saharan African countries, has begun to emerge in the United States. While the first outbreak in the United States was detected in 2003, there was a resurgence in 2022 after a long period of sporadic cases [1,2]. The monkeypox virus is a member of the Orthopoxvirus genus within the Poxviridae family [3]. The disease presents similarly to a mild version of smallpox, starting with a prodromal illness with nonspecific viral symptoms such as fever, chills, lethargy, lymphadenopathy, back pain, and myalgias. The fever precedes the rash, which appears first on the face and spreads in a cephalocaudal pattern with morphological changes consisting of macular, papular, and vesicular stages [4]. Involvement of mucosal surfaces such as the pharynx, conjunctivae, and genitalia can occur. The rarity of monkeypox has prevented any epidemiological studies regarding the ocular involvement of the disease. When the eye is involved, monkeypox can cause conjunctivitis, blepharitis, and corneal damage, along with focal cutaneous lesions surrounding the eye [5]. The most common ocular manifestation of the virus is the characteristic rash in the periorbital and orbital areas [6]. While different agents have been tried and investigated, there is no specific treatment for monkeypox. Supportive care and treating any secondary bacterial infections is the preferred recommendation [4]. Here, we describe a case of a female patient who presented initially with signs and symptoms consistent with periorbital cellulitis and developed the characteristic disseminated monkeypox rash after her admission to the hospital. 

## Case presentation

A 38-year-old female with a self-reported past medical history of chlamydia infection and no chronic illnesses presented to the hospital as a transfer for management of periorbital cellulitis. The patient presented with elevated blood pressure along with significant erythema and swelling on the right upper and lower eyelid. Symptoms started one week prior with periorbital swelling, erythema, lacrimation, and pain. Prior to transfer, she was seen in the emergency room at the transferring facility on day three of symptoms, where a CT of the orbits showed evidence of right periorbital cellulitis, including peripherally enhancing loculated air and fluid measuring 5 mm in thickness. There was no evidence of orbital cellulitis observed on the imaging. The patient was empirically treated with vancomycin, ceftriaxone, and metronidazole in the emergency room. Upon admission to the transferring hospital, the treatment was switched to piperacillin/tazobactam, along with analgesics and antihypertensives. On exam, the patient was afebrile and alert but had swelling, warmth, and erythema extending beyond the periorbital skin to the right side of the face. She was unable to open her right eye due to throbbing pain and swelling.

**Figure 1:**
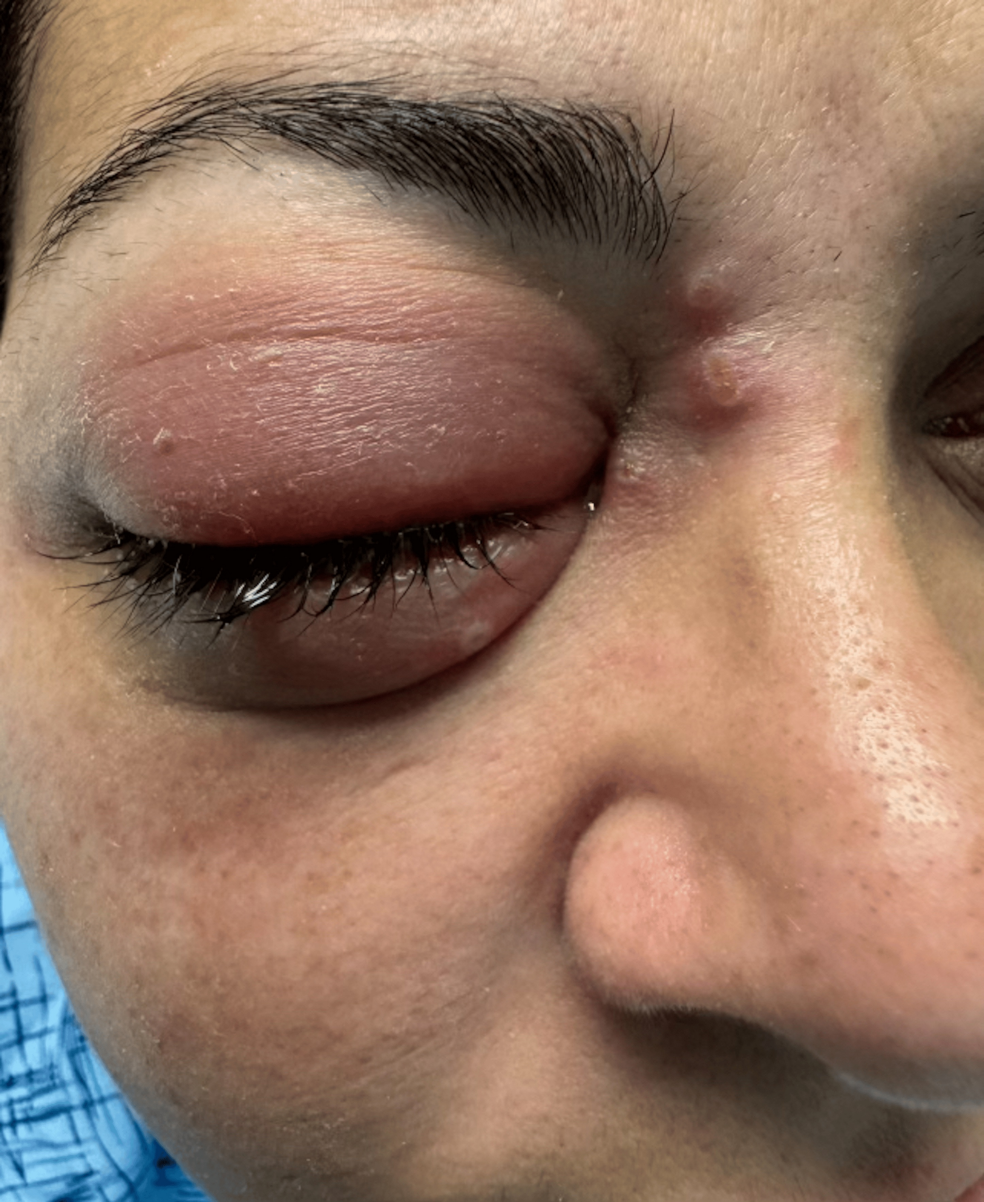
Erythema and marked swelling of the right upper and lower eyelid with conjunctival tearing, accompanied by four vesicles located inferomedial to the eye

**Figure 2:**
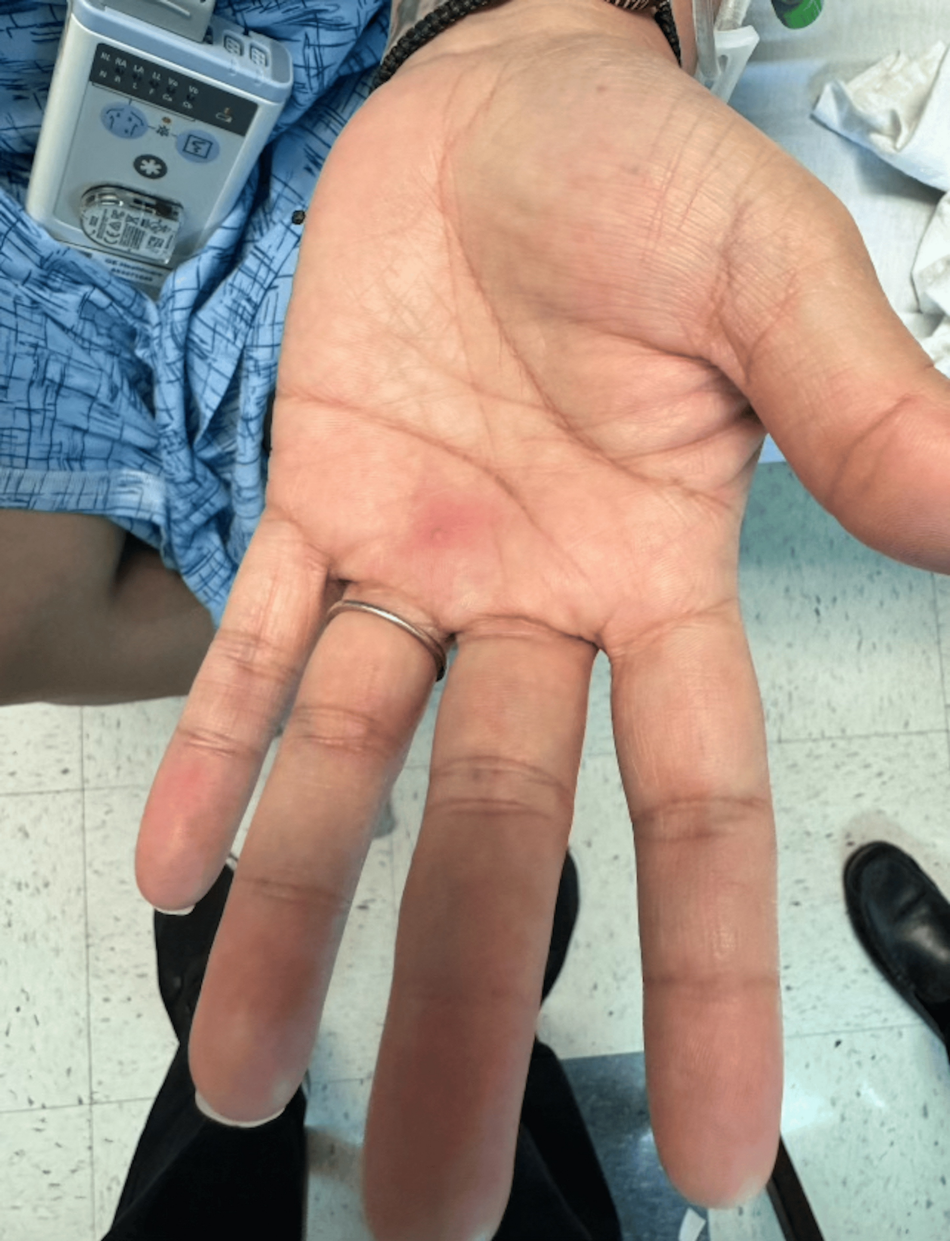
Single vesicle on the palm with poorly demarcated surrounding erythema

**Figure 3:**
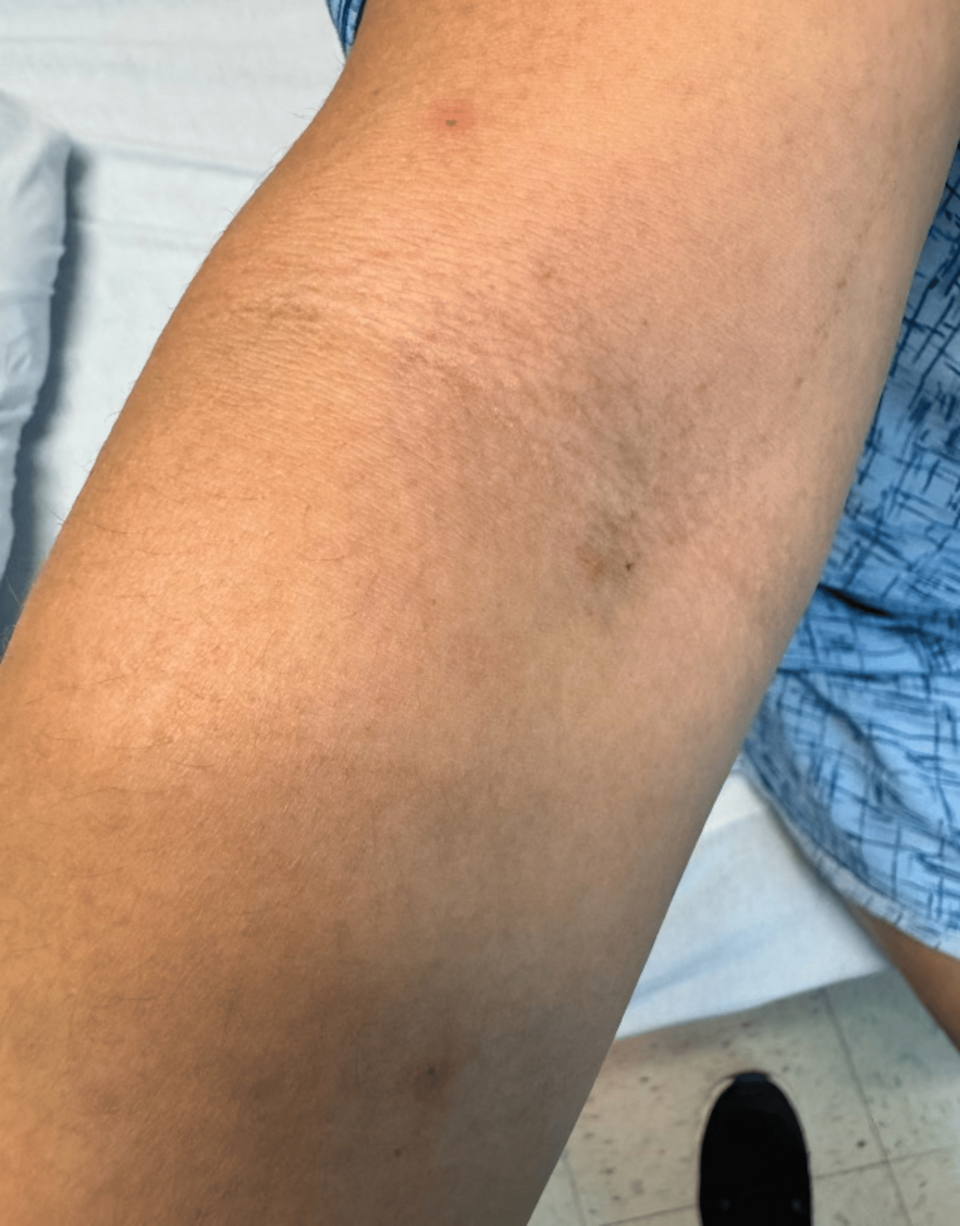
Three 1 mm crusted papules with residual surrounding erythema located on the right ventral upper arm and forearm

An MRI of the face and neck was obtained, which reported pronounced periorbital cellulitis. Over the course of her hospitalization, she developed erythematous papules along the medial periorbital skin on the affected side (Figure 1) along with similar scattered vesicular lesions on her upper extremities (Figures 2-3), lower extremities, and abdomen. The lesions ranged from 2-3 mm in size and appeared vesicular with an overlying crust. A viral polymerase chain reaction (PCR) for herpes simplex virus (HSV) and varicella zoster was obtained as well as a human immunodeficiency virus (HIV) test, and the patient was started on acyclovir along with switching her antibiotic to ampicillin/sulbactam. While the patient improved clinically with the new treatment regimen, her skin lesions persisted throughout her hospital course. After she tested negative for HSV, varicella zoster, and HIV, a monkeypox PCR was ordered and she was discharged on oral valacyclovir and amoxicillin/clavulanate. After her discharge, her PCR results reported positive for monkeypox, and the local health department was informed. When the patient was informed of her diagnosis, she reported continued improvement in her skin lesions and overall condition and agreed to follow up with ophthalmology and infectious disease for outpatient evaluation and management. 

**Figure 4:**
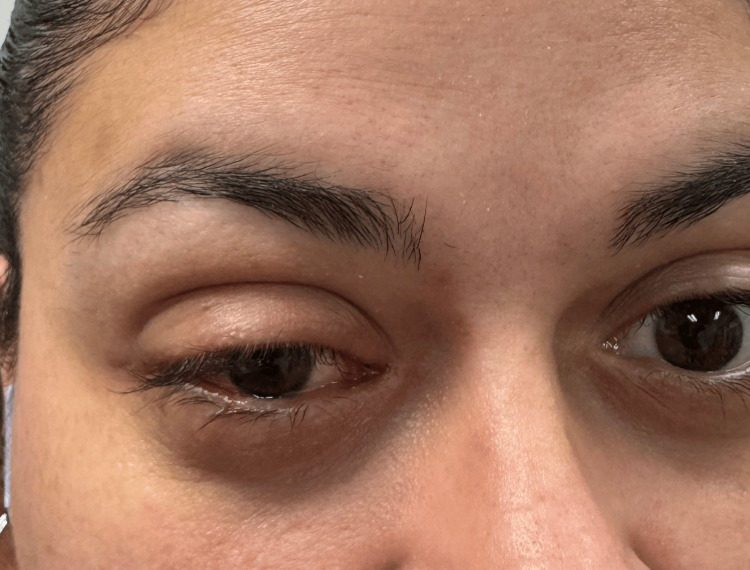
Image of the affected eye four weeks after discharge from the hospital. Post-inflammatory pigment change present at the location of previous eruptions with some residual swelling in the upper eyelid

The patient followed up as an outpatient with an infectious disease specialist three weeks after discharge. She completed her prescribed antibiotic and antiviral regimens and had no residual periorbital erythema at this visit. Her vesicular and papular eruptions (Figures 1-3) had also improved, although she had some persistent papules on the dorsal hands bilaterally. She remained afebrile throughout her illness, had no other symptoms, and did not develop any new vesicles since completing her oral treatment. Figure 4 shows how her skin healed four weeks after she was discharged from the hospital.

## Discussion

Monkeypox is a multifaceted zoonotic infectious disease that impacts various organ systems, presenting a diagnostic challenge for clinicians due to its unfamiliar nature. Given the patient’s initial presentation, periorbital cellulitis was the leading diagnosis upon admission. Most cases of periorbital cellulitis result from the local spread of rhinosinusitis or infections caused by local trauma, with *Staphylococcus aureus*, *Streptococcus pneumoniae*, and *Streptococcus pyogenes* being the most isolated pathogens. Monkeypox manifests in humans similarly to smallpox but generally follows a milder course. Monkeypox has an incubation period of 5-21 days and initially presents with a viral prodrome lasting 1-5 days, primarily characterized by fever. Other nonspecific flu-like symptoms can also occur, including headache, muscle pain, fatigue, swollen lymph nodes, chills, and sweating [3,7,8]. After the flu-like symptoms subside, a maculopapular rash emerges and spreads centrifugally, often involving mucosal membranes such as the eyes and anogenital areas but is most prominently located on the face (95% of cases) [7,9]. Like smallpox, the skin lesions evolve in appearance from macules to papules, vesicles, and pustules before crusting over and ultimately resolving [7]. The rash is usually observed to be in various stages of progression simultaneously in a patient. It can last up to four weeks before the skin lesions form scabs and resolve. It is atypical for the rash to appear before the fever or for the prodrome phase to be entirely skipped [8]. Extra-integumentary manifestations can occur in the upper respiratory tract as pharyngitis or tonsillitis, and in the hepatobiliary tract as hepatic transaminitis. Genitourinary involvement is commonly observed in nonendemic areas and is often the reason for seeking medical attention, particularly in men who have sex with men (MSM), a high-risk group. Typical symptoms include lesions on the external genitalia, penile edema, and tender regional lymphadenopathy [3]. 

Damavandi et al. conducted a literature review in 2022 of all ocular manifestations of the monkeypox virus reported to date [8]. Outbreaks of monkeypox that occurred prior to the 2022 outbreak most frequently reported blepharitis and conjunctivitis as the most common ocular manifestations. In the United States, blepharitis was reported in 9% of patients in the 2003 outbreak. During the 2022 outbreak, periorbital involvement included eyelid and surrounding characteristic skin lesions, periorbital edema, and preseptal cellulitis accompanied by or secondary to conjunctivitis and blepharitis. The specific case involving preseptal cellulitis resulted from direct inoculation when the patient rubbed his right eye with lesions present on the penis, abdomen, and wrist [10]. A case was reported in San Francisco, California involving an HIV-negative individual with a background of chronic lymphocytic leukemia; the patient presented solely with right eye itching and nasal scleral erythema, without fever, rash, or lymphadenopathy. After treatment with topical erythromycin, the patient suffered vision impairment and was diagnosed with preseptal cellulitis. Treatment involved a regimen of trimethoprim/sulfamethoxazole in combination with amoxicillin/clavulanic acid. The case progressed to nasal scleral necrosis, scleritis, corneal epithelial sloughing, keratitis, and corneal limbitis [11]. The patient later admitted to high-risk sexual activities outside of penetrative intercourse, describing an encounter with semen deposition into his right eye two weeks before the onset of symptoms. Overall, periorbital swelling in cases of the monkeypox virus occurs after the development of skin lesions or is accompanied by characteristic skin lesions in the periorbital area. Literature involving the eye and periorbital area on monkeypox infection is focused mainly on ophthalmic involvement and complications, due to the severe impact on vision and quality of life. 

While there is no specific treatment for human monkeypox infection, there are some viral agents that have been studied. Cidofovir, used for cytomegalovirus retinitis in HIV patients, has been studied in nonhuman primates and mouse models with the conclusion that it may benefit in managing human monkeypox infection if administered prior to the onset of cutaneous symptoms [3]. Brincidofovir, an analog of cidofovir, has been studied to a small extent [6]. It was used to treat three infections of monkeypox in 2018 in the United Kingdom, but treatment was terminated early due to hepatotoxicity [3]. The most efficacious antiviral agent studied is tecovirimat (Tpoxx®), which showed success in nonhuman primate, prairie dog, and mouse models. Tpoxx® is a low-molecular-weight orthopoxvirus inhibitor developed to treat smallpox and was licensed for the treatment of monkeypox in 2022 by the European Medicines Agency [6]. Oral Tpoxx® was used in conjunction with topical trifluridine and ophthalmic betadine washes for four months in the case mentioned above in San Francisco [11]. However, worsening vision led to inpatient treatment with intravenous Tpoxx® and cidofovir for three weeks which failed to improve ocular disease. Further, the Modified Vaccinia virus Ankara (MVA) vaccine, a third-generation vaccine previously approved for smallpox prevention, has been recommended by the Centers for Disease Control and Prevention and the Advisory Committee on Immunization Practices for pre- and post-exposure prophylaxis for human monkeypox infection [6]. 

A survey of the existing literature did not reveal reports specifically discussing periorbital cellulitis as the initial presentation of monkeypox before the development of skin lesions or flu-like symptoms. Periorbital cellulitis, although common, shares overlapping clinical features with other conditions, necessitating a comprehensive differential diagnosis. In regions where monkeypox is endemic or during outbreaks, healthcare providers should maintain a high index of suspicion for unusual infectious etiologies, even in immunocompetent patients. According to the Florida Department of Health, there have only been 16 reported confirmed cases since the start of 2010 in the county where the patient was seen, and over 1800 confirmed cases throughout Florida since 2020. Most cases are in larger counties such as Broward, Miami-Dade, Hillsborough, Orange, and Pinellas County [12].

## Conclusions

This case is unique in its atypical presentation of periorbital cellulitis preceding the development of skin lesions, stressing the importance of keeping monkeypox on the list of differential diagnoses when evaluating a patient presenting in a similar way. A detailed examination of oral, ocular, and anogenital mucosal membranes is necessary for a comprehensive workup when considering communicable etiologies. 
